# Rubella myopericarditis and cardiac tamponade: a case report

**DOI:** 10.1093/omcr/omae130

**Published:** 2024-11-20

**Authors:** Yohei Ishibashi, Yoshito Nojiri, Yosuke Takahashi, Shinya Takahashi, Nobuaki Fukuda, Shitoshi Hiroi

**Affiliations:** Department of Cardiology, NHO Takasaki General Medical Center, Takasaki, Japan; Department of Cardiology, NHO Takasaki General Medical Center, Takasaki, Japan; Department of Cardiology, NHO Takasaki General Medical Center, Takasaki, Japan; Department of Cardiology, NHO Takasaki General Medical Center, Takasaki, Japan; Department of Cardiology, NHO Takasaki General Medical Center, Takasaki, Japan; Department of Cardiology, NHO Takasaki General Medical Center, Takasaki, Japan

**Keywords:** pericarditis, myocarditis, perimyocarditis, viral pericarditis, pericardial effusion, pericardiocentesis

## Abstract

A 20-year-old male patient with ulcerative proctitis presented with a fever and chest pain. He was diagnosed with rubella-associated myopericarditis due to pericardial rub, elevated troponin I, ST elevation, and positive rubella-immunoglobulin M. The patient subsequently developed cardiac tamponade but responded well to pericardial drainage and antiinflammatory therapy. Notably, he lacked the classic rubella rash and lymphadenopathy. This case highlights the rare but potential complication of rubella-induced myopericarditis with tamponade, and the importance of considering this diagnosis in the absence of typical rubella symptoms.

## Introduction

Rubella cases are declining worldwide due to widespread vaccination, although the disease has not been eradicated [1]. In this regard, Japan has experienced occasional rubella outbreaks despite improvements in its vaccination program [2]. While rubella infection is typically mild, causing symptoms such as fever, rash, and swollen lymph nodes, complications can include arthritis, thrombocytopenic purpura, and encephalitis [3]. However, cardiac complications are rarely reported [4].

Acute pericarditis is an inflammatory condition of the pericardium. Moreover, myopericarditis is a specific type of pericarditis that also involves myocardial injury, but without dysfunction of the left ventricle pumping ability [5]. Pericarditis may have various causes, including viral, bacterial, tuberculous, drug-induced, eosinophilic, autoimmune, metabolic, and idiopathic (unknown) origins. Viral infections are a common cause, with coxsackievirus, echovirus, and parvovirus B19 being the most frequently responsible for the condition. Rubella, however, is a rare cause of pericarditis [6].

Herein, we report a rare case of rubella-associated myopericarditis with cardiac tamponade, presenting without the typical symptoms of rubella.

## Case report

**Figure 1:**
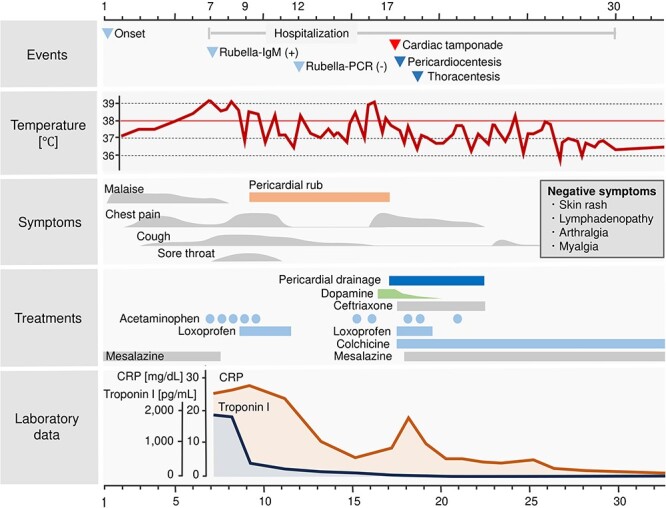
Clinical course. Course of body temperature, symptoms, laboratory data, and treatments. CRP: C-reactive protein. Ig: immunoglobulin. PCR: polymerase chain reaction.

**Figure 2:**
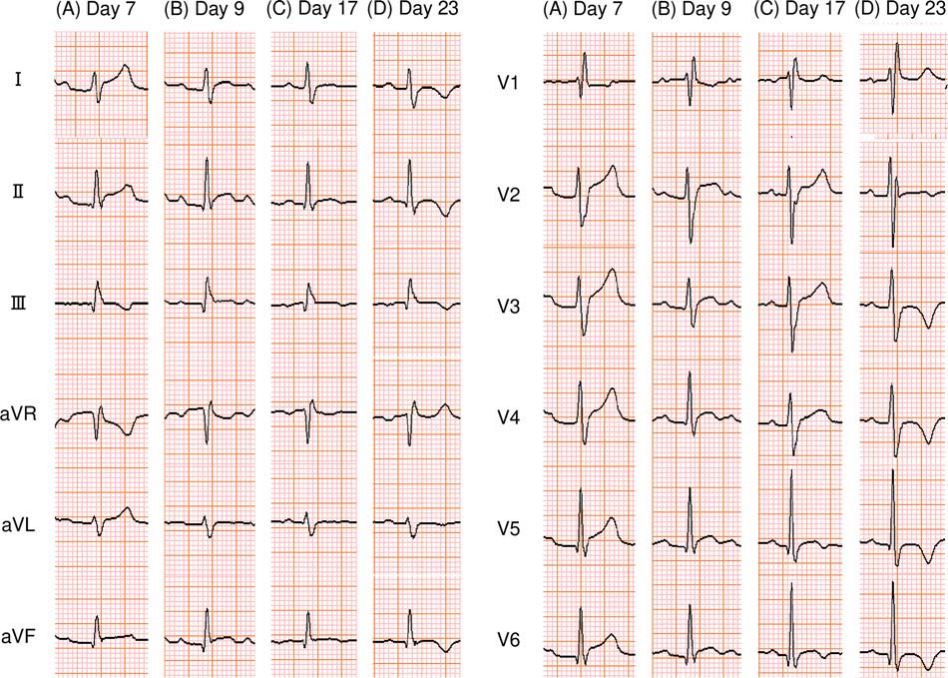
12-lead ECG. PR depression and ST elevation were observed (A and B), then became less noticeable (C), and negative T-waves appeared (D). ECG: electrocardiogram.

A 20-year-old male patient presented with malaise and a temperature of 37.2°C. He subsequently developed chest pain and a cough (Fig. 1). He visited a neighboring hospital on day 7 and was then transferred to our hospital because of ST elevation on an electrocardiogram (ECG) (Fig. 2A).

He was previously diagnosed with ulcerative proctitis and stabilized with mesalazine at 2700 mg daily for 1 year. He had been vaccinated against rubella at ages 3 and 5.

**Figure 3:**
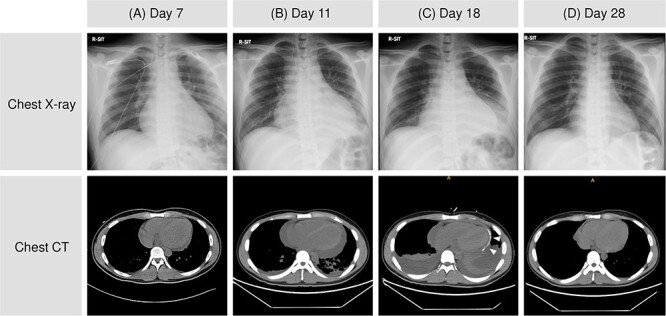
Chest X-ray and CT. Cardiac enlargement and pericardial effusion worsened (A and B) and pleural effusions became apparent after pericardial drainage (arrowheads) (C). Pericardial and pleural effusion then improved (D). CT: computed tomography.

**Figure 4:**
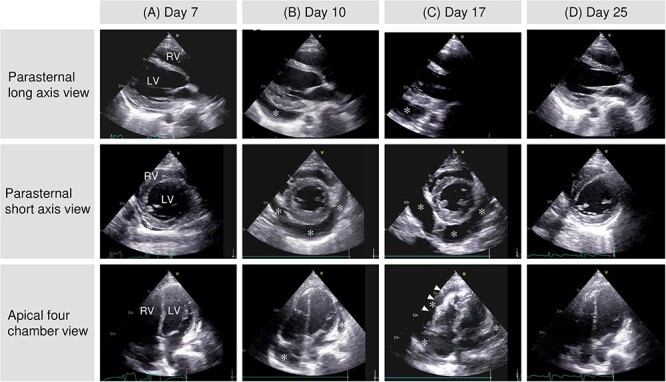
TTE findings. A gradual increase in pericardial effusion (*) was observed (A and B). This accumulation of fluid eventually led to the collapse of the right ventricle (RV) (arrowheads), a sign of cardiac tamponade (C). Pericardial effusion decreased following drainage (D). The LV contraction remained normal throughout. LV: left ventricle, RV: right ventricle, TTE: transthoracic echocardiography.

The patient was alert and oriented, with a temperature of 39.3°C, pulse rate of 78/min, blood pressure of 123/76 mmHg, respiratory rate of 26/min, and oxygen saturation of 100% without supplemental oxygen. Physical examination of the chest revealed no abnormalities on chest auscultation, and there was no skin rash. Blood tests revealed an elevated number of white blood cells, C-reactive protein (CRP), troponin I, and the N-terminal prohormone of brain natriuretic peptide (NT-proBNP), but not in creatine kinase (Table 1). Chest X-ray and computed tomography (CT) revealed an enlarged heart (Fig. 3A). Transthoracic echocardiography (TTE) revealed a small pericardial effusion and no left ventricular dysfunction (Fig. 4A). He was diagnosed with acute myopericarditis. Suspected drug-induced myopericarditis, mesalazine was discontinued. Loxoprofen 180 mg daily was administered minimally depending on chest pain, considering the risk of aggravating ulcerative proctitis and myocarditis (Fig. 1).

**Table 1:** Results of the representative blood tests on admission

**Laboratory tests**	**Results**	**(Reference)**
Hemoglobin, [g/dl]	11.5	(13.7–16.8)
White blood cell count, [/μl]	14 100	(4250–5550)
(Neutrophil 71%, Eosinophil 0%, Basophil 0%, Monocyte 12%, Lymphocyte 17%)		
Platelet count, [/μl]	346 000	(137 000–501 000)
Fibrinogen, [mg/dl]	696	(200–400)
Prothrombin time-international normalized ratio	1.36	(0.85–1.15)
Activated partial thromboplastin time, [sec]	30.3	(25.0–40.0)
D-dimer, [μg/ml]	2.7	(<1.0)
Total protein, [g/dl]	7.0	(6.6–8.1)
Albumin, [g/dl]	2.9	(4.1–5.1)
Aspartate aminotransferase, [U/l]	26	(13–30)
Alanine aminotransferase, [U/l]	25	(10–42)
Lactate dehydrogenase, [U/l]	176	(124–222)
Blood urea nitrogen, [mg/dl]	10.1	(8.0–20.0)
Creatinine, [mg/dl]	0.95	(0.65–1.07)
Creatine kinase, [U/l]	129	(59–248)
Creatine kinase-MB, [U/l]	<4	(<12)
Amylase, [U/l]	37	(44–132)
Glucose, [mg/dl]	106	(73–109)
Sodium, [mEql/l]	134	(138–145)
Potassium, [mEq/l]	4.4	(3.6–4.8)
Chloride, [mEq/l]	96	(101–108)
C-reactive protein, [mg/dl]	24.26	(<0.14)
NT-proBNP, [pg/ml]	1406	(<125)
Troponin I, [pg/ml]	1888	(<26.2)

NT-proBNP: N-terminal prohormone of brain natriuretic peptide.

**Figure 5:**
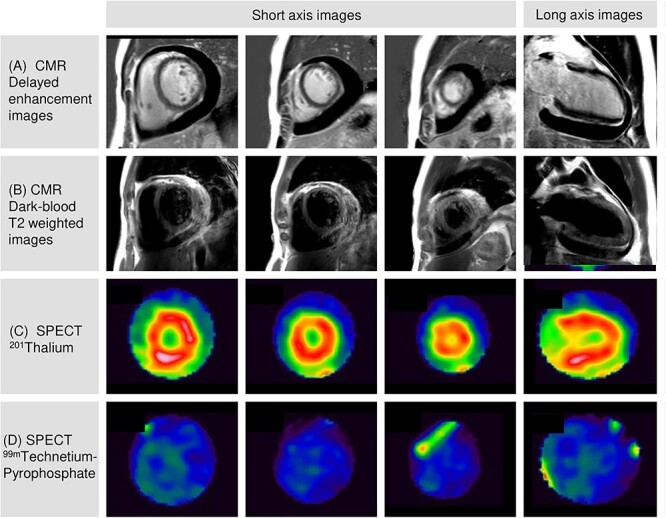
CMR and SPECT. CMR revealed no findings of delayed enhancement (A) and T2-weighted high signal (B) in the myocardium and pericardium. ^201^Thalium SPECT demonstrated normal myocardial perfusion (C). ^99m^Technetium-pyrophosphate did not accumulate in the myocardium (D). CMR: cardiac magnetic resonance; SPECT: single-photon emission computed tomography.

Screening tests revealed a possible acute rubella infection, with a positive rubella-immunoglobulin (Ig) M test. However, rubella polymerase chain reaction (PCR) tests on blood, urine, and throat swabs collected on day 12 were negative (Table 2). On day 12, cardiac magnetic resonance (CMR) showed no obvious signs of fibrosis, edema, or inflammation in the myocardium or pericardium (Fig. 5A and B). Additionary, a single-photon emission computed tomography (SPECT) on day 13 revealed normal perfusion (Fig. 5C) and did not detect any evidence of myocardial infarction or necrosis (Fig. 5D).

**Table 2:** Results of the investigation for etiology of myopericarditis

**Possible etiologies**	**Results**
Viral investigation	
Adenovirus	No significant increase in antibody titer on days 7, 21
Echovirus type-4, 6, 9, 11	No significant increase in antibody titer on days 7, 21
Coxsackie virus type-A5, A9, A16, B1, B2, B3, B4, B5	No significant increase in antibody titer on days 7, 21
Epstein–Barr virus	EB-VCA-IgM negative
Cytomegalovirus	IgM negative, IgG positive
Parvovirus B19	IgM negative
Mumps virus	IgM negative, IgG positive
Measles virus	IgM negative, IgG positive
Rubella virus	IgM positive of 3.19 (<0.80), IgG positive of 9.2 (<2.0) (day 7) IgM negative of 0.25 (<0.80), IgG positive of 13.9 (<2.0) (day 21) IgM and IgG were measured using the Enzyme Immunoassay method. Blood, urine, and throat swab (day 12), pericardial fluid (day 17), and pleural fluid (day18) PCR negative
SARS-CoV-2	PCR negative
Influenza virus type A, B	Antibody negative
Hepatitis A	IgM negative
Hepatitis B	HBs antigen of 0.03 mIU/ml (<0.05)
Hepatitis C	Antibody of 0.11 (<1.00)
HIV	HIV 1p24 antigen/HIV antibody combo assay negative
Bacteriological investigation	
Culture tests	Blood, urine, and sputum (day 7), pericardial fluid (day 17), and pleural fluid (day 18) negative
Procalcitonin	0.21 (<0.50) ng/ml
*Treponema pallidum*	Antibody negative
T-SPOT. TB test	Negative
Mycological investigation	
β-D-glucan	16.6 (<20.0) pg/ml
Autoimmunological investigation	
Rheumatoid factor	17.8 (<15) IU/ml
Antinuclear antibody	<40 (<40) titer

EB-VCA: Epstein–Barr virus capsid antigen; HBs: hepatitis B surface; HIV: human immunodeficiency virus; Ig: immunoglobulin; PCR: polymerase chain reaction; SARS-CoV-2: severe acute respiratory syndrome coronavirus 2; T-SPOT. TB: T cell spot test for tuberculosis. Numbers in parentheses after inspection data represent reference values.

**Figure 6:**
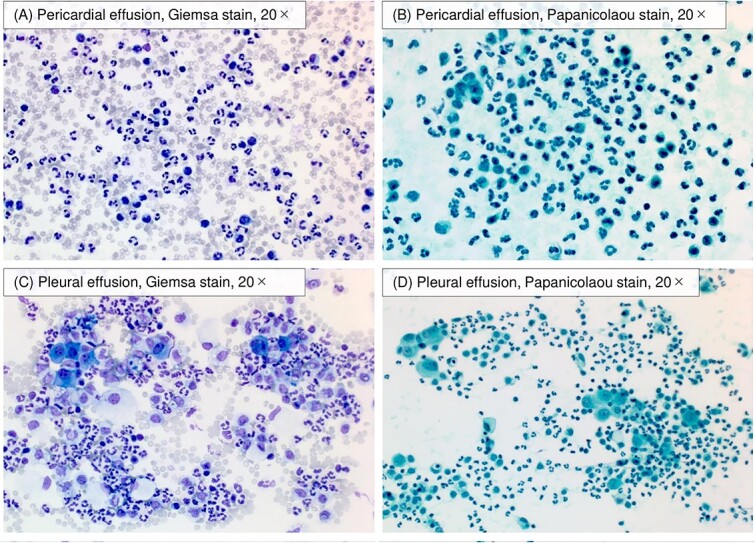
Cytological images of pericardial and pleural effusion. Cytology of pericardial effusion revealed a neutrophil-dominant inflammatory pattern against a background of red blood cells (A and B). Cytology of pleural effusion demonstrated the inflammatory pattern (C and D).

A pericardial rub was heard starting on day 9 (Fig. 1). Chest CT and TTE also showed an increase in pericardial effusion (Figs 3B and4B). However, his chest pain, fever, and CRP level gradually improved. On day 17, chest pain worsened, and blood pressure dropped (Fig. 1). TTE revealed right ventricular collapse, with a diagnosis of cardiac tamponade (Fig. 4C). Dopamine was administered at a maximum of 10 μg/min/kg, and pericardiocentesis and drainage were performed. The pericardial effusion was pale, bloody exudative inflammatory fluid (Table 3, Fig. 6A and B). The CRP level was elevated again, and the bilateral pleural effusion increased (Fig. 3C). Inflammatory fluid obtained from the right thoracentesis on day 18 indicated a complication of bilateral pleurisy (Table 3, Fig. 6C and D). Loxoprofen at 180 mg daily and colchicine at 1 mg daily were initiated, and mesalazine was restarted. Loxoprofen was then discontinued the next day because of worsening bloody stools. Ceftriaxone at 2 mg daily was temporarily administered, while the blood culture was negative. Colchicine was continued, and pericardial and pleural effusions gradually decreased (Figs 3D and 4D).

**Table 3:** Results of the tests of pericardial effusion and pleural effusion

**Tests**	**Pericardial effusion**	**Pleural effusion**
Collection day	Day 17	Day 18
Appearance	Pale-bloody	Brownish-yellow
Turbidity	+	2+
Specific gravity	1.039	1.027
Rivalta test	Positive	Negative
Red blood cell count, [/μl]	113 000	21 000
White blood cell count, [/μl]	13 040	8145
Mononuclear cells, [%]	15.0	32.3
Polynuclear cells, [%]	85.0	67.7
Total protein, [g/dl]	5.6	3.7
Ratio to serum total protein	0.90	0.63
Lactate dehydrogenase, [U/l]	682	194
Ratio to serum lactate dehydrogenase	3.07	1.26
Amylase, [IU/l]	38	21
Adenosine deaminase, [IU/l]	25.0	13.0
Sodium, [mmol/l]	134	135
Potassium, [mmol/l]	4.0	4.0
Chloride, [mmol/l]	103	106
C-reactive protein, [mg/dl]	5.26	5.22
General bacterial culture	Negative	Negative
Acid-fast bacilli culture	Negative	Negative
Cytology	Class II, inflammation	Class II, inflammation
Rubella-PCR	Negative	Negative

PCR: polymerase chain reaction.

By day 21, rubella-IgM had become negative, while rubella-IgG levels had risen. No other tests identified a potential cause for the perimyocarditis (Table 2). Despite negative rubella-PCR results in blood, urine, pharyngeal swab, pericardial fluid, and pleural fluid, the clinical picture and serological findings (positive rubella-IgM followed by rising IgG) supported a diagnosis of rubella-associated myopericarditis.

The patient was discharged home on day 30 (Fig. 1). He continued taking colchicine for 3 months with no return of perimyocarditis or development of complications like constrictive pericarditis.

## Discussion

We report a rare case of acute rubella-associated myopericarditis with cardiac tamponade. Diagnosing rubella as the cause was challenging due to the absence of typical rubella symptoms, but positive rubella-IgM in screening tests ultimately supported this conclusion.

Myopericarditis is defined as pericarditis with myocardial injury without left ventricular dysfunction [5]. The most prominent etiology is viral infection, but rubella is rare [6]. To date, 10 cases of cardiac complications of acquired rubella have been reported, including 6 cases of pericarditis or myocarditis, but none were complicated cardiac tamponade [4]. This was a rare case of rubella-associated myopericarditis with cardiac tamponade.

Most viral pericarditis is not severe, but poor prognostic factors, including failure of initial treatment and large pericardial effusion, have been noted [7]. The guideline recommends nonsteroidal antiinflammatory drugs (NSAIDs) and colchicine for treating pericarditis [5]. However, the use of NSAIDs and colchicine is controversial in cases of complicated myocarditis [7]. Furthermore, NSAIDs have a risk of aggravating ulcerative colitis [8], and colchicine has gastrointestinal side effects [9]. The use of corticosteroids is not recommended for viral pericarditis [5]. Therefore, the initial antiinflammatory treatment for myopericarditis was eventually insufficient in this case. This was considered a factor that complicated cardiac tamponade.

Pericarditis is a relatively prevalent complication of ulcerative colitis including ulcerative proctitis. One factor is immune-mediated secondary to autoantigen exposure, and the other is the cardiotoxic effects of drugs, including mesalazine. Mesalazine-induced pericarditis is most prevalent within 2 weeks of drug initiation [10]. In this case, the immunological mechanism associated with ulcerative colitis might be a factor of perimyocarditis. However, this case differs from previous reports due to the one-year duration of mesalazine use. Furthermore, even after the reintroduction of mesalazine, myopericarditis improved and did not recur. These factors suggest that mesalazine is unlikely to be the causative agent in this case.

Rubella generally causes fever, skin rash, and lymphadenopathy, but 25%–50% of cases are asymptomatic. While the efficacy of the vaccine is generally high, individual responses can vary [11]. It has been reported that even after receiving two doses of the vaccine, some individuals may not develop sufficient neutralizing antibodies [12]. In fact, Japan’s National Institute of Infectious Diseases has reported that 1%–2% of rubella cases occur in individuals who have received two doses of the vaccine [13]. Complications of rubella excluding congenital rubella syndrome include arthritis, thrombocytopenic purpura, and encephalitis, but cardiac complications are rare [3]. Of the 10 cases of cardiac complications from rubella, 3 did not present with a rash [4]. This case had no rash or lymphadenopathy, was not surrounded by individuals infected with rubella, and had a history of vaccination, making the clinical diagnosis of rubella difficult.

Serologic testing for the etiology of viral pericarditis is not recommended [5]. Therefore, viral testing, including testing for rubella, is not performed usually in actual cases of pericarditis. Thus, rubella may be overlooked in cases of idiopathic pericarditis. Although rubella has no specific treatment, but its diagnosis is highly significant for countermeasures against the surrounding infection. Perhaps, it should be the subject of screening for pericarditis, especially in areas where rubella has not been eradicated.

For the diagnosis of rubella, rubella-IgM and PCR are recommended [14]. However, rubella-IgM is known to cross-react with enteroviruses, adenoviruses, parvovirus B19, and rheumatoid factor, leading to false positives [15]. In this case, screening tests for viruses that could potentially cause cross-reactivity returned negative results, although the possibility of cross-reactivity with viruses that were not tested for cannot be completely ruled out. Furthermore, rubella-IgM initially increased but became negative three weeks later. A sustained increase in rubella-IgM following rubella vaccination was ruled out. The rise in rubella-IgG observed three weeks later is consistent with acute infection. However, the antibody level increase was less than twofold, which is atypical for acute rubella infection. This may be due to the limitations of the Enzyme Immunoassay method’s quantitativeness or the timing of the test. Additionally, rubella-PCR is reported to have lower sensitivity than rubella-IgM [16]. In this case, the negative rubella-PCR result was thought to be due to delayed testing or the lower sensitivity of the rubella-PCR. Therefore, while caution is required in definitively concluding that this case was due to acute rubella infection, we ultimately diagnosed the patient with rubella-associated myopericarditis.

The primary mechanism of the cardiac complications of rubella was considered an excessive immune response rather than direct viral action [4]. The negative rubella-PCR results for pericardial and pleural fluid in this case may support that the primary mechanism of rubella-associated myopericarditis was immunological overreaction after the viral damage.

In conclusion, this was a rare presentation of rubella-associated myopericarditis, complicated by cardiac tamponade. Rubella demonstrated no specific symptoms, making its clinical diagnosis difficult.
